# Projections of wildfire weather danger in the Canary Islands

**DOI:** 10.1038/s41598-022-12132-5

**Published:** 2022-05-16

**Authors:** J. Carrillo, J. C. Pérez, F. J. Expósito, J. P. Díaz, A. González

**Affiliations:** Grupo de Observación de la Tierra y la Atmósfera (GOTA), Universidad de La Laguna (ULL), San Cristóbal de La Laguna, Canary Islands Spain

**Keywords:** Climate change, Climate and Earth system modelling, Climate-change impacts, Climate-change mitigation, Projection and prediction

## Abstract

Climate change is expected to enhance weather conditions prone to wildfires. Climate regionalized projections for the Canary Islands were performed, using as boundary conditions some of the results provided by the Coupled Model Intercomparison Project (CMIP5) initiative, and covering the recent past (1980–2009) and future (2070–2099) periods, under two Representative Concentration Pathways, 4.5 and 8.5. All fire risk indicators derived from the Canadian Forest Fire Weather Index (FWI) are foreseen to worsen by the end of the century. The fire season could increase its length up to 75 days per year, being more noticeable as altitude increases. The extreme risk days (FWI > 60) show an average increase of 58%, reaching 12 days a year, and the area with high risk could increase by 44%. Analyzing the contribution of the different meteorological variables, it is observed that the main parameter in the fire danger index result is the temperature (currently weights 46%). However, in the future, the importance of precipitation will increase, since the rainfall reduction in some areas could reach 41%. The high dependence of the expected changes on land height, and the small size of the islands, demonstrates the necessity of using high-resolution climate regionalizations.

## Introduction

Recent publications strengthen the evidence that climate change increases the occurrence and severity of fire weather, that is, periods with a high fire risk due to a combination of high temperatures, low humidity, low rainfall and often high winds, making suppression efforts more difficult^1^. A strong increase in the global frequency of long fire weather seasons has been observed^2^, and the burnt area by wildfires has been found to be increasing in the western US and southeast Australia^1^. Future projections in large areas suggest that climate change may cause fire-intensifying in Europe^3,4^, southeastern Australia^5^, Russia^6^ or Central Asia^7^ at the end of the century.

Wildfires are analyzed from two perspectives: fire danger, defined by indices, and their impact, mainly reflected in the burnt area^8–12^. Concerning the indices, the Fire Weather Index (FWI)^13–18^ is a numeric rating of fire intensity and is used as an index of fire danger. Its based on the four variables most widely analyzed due to their impact on the number of wildfires and their severity, that is, temperature, humidity, wind, and precipitation. The European Forest Fire Information System, EFFIS network, adopted the FWI as the method to assess the fire danger level in a harmonized way throughout Europe and the Spanish State Meteorology Agency (AEMET) uses it for its operational fire alert system. The FWI is not defined by a linear equation, but a set of conditions to be scored. This makes it difficult to analyze the sensitivity of the parameters and their impact on the FWI, also considering that they are not independent terms. In previous studies, high temperature has been identified as the most important variable affecting fire danger, reducing fuel moisture by evapotranspiration and increasing lightning activity^19^. Besides, temperature and humidity influence the type and availability of fuel and fuel moisture will determine its flammability^19^. Nevertheless, other studies conclude that FWI is most sensitive to wind speed, then secondly to relative humidity, and thirdly to temperature^15^ or, on the contrary, that average precipitation of the warmest quarter has the strongest influence on the fire occurrence^20^. Knowing the weight of each of the previous parameters in future fire risk could help in the design of mitigation policies (e.g. choice of plant species, identification of high-risk areas in land planning, etc.), and promote healthy ecosystems.

The unevenness of the terrain also impacts the spread of fire in blazes, and it generates a subsequent problem, the possibility of landslides. The soil that the forests protect is exposed to powerful displacements by losing the natural support that the trees provide, increasing this risk^21^. Furthermore, wildfires have a direct impact on the ecosystems and on society: risk to human lives, damage to infrastructure, and economy, e. g.: costs of suppression (personnel, equipment, logistics, etc.), and from the recovery process^22^. Fires are also a significant source of pollution, including carbon gases, particulate matter, and volatile organic compounds^23^.

All these consequences of wildfires are of great importance in archipelagos such as the Canary Islands, with a complex orography and a very sensitive ecosystem full of endemic species^24^. During the last decades, the Canary Islands have been affected by numerous fires. Between 1983–2009, estimates of the archipelago’s burnt area exceeded 76,000 ha. Some years have been especially dramatic, such as 2007 in which 35,000 ha were burnt, specifically in La Palma, Tenerife, and Gran Canaria. On the island of Gran Canaria, in a single fire declared in July 2007, nearly 18,700 ha were burnt, 12% of the island’s surface. Later, in 2019, a fire devastated 10,000 ha on the same island; the local administration valued from 15 million euros for the expenditure to alleviate the damage from the fire. A historical study carried out with dendrochronological methods in *Pinus canariensis* has concluded that over the last several decades fire suppression has eliminated all but the largest higher intensity wildfires, establishing a new fire regime^25^. The mean annual percentage of the surface of the islands devastated by fires, 0.38% in the period 1983–2009, is very similar to that of the Mediterranean countries, which are the most affected in Europe (see Supplementary Table S1).

Nevertheless, this fire regime is expected to modify under climate change. To understand the risks derived from the changing conditions, climate projections have demonstrated to be efficient tools to evaluate the effects of the new possible scenarios. In this sense, results from Global Climate Models (GCM) simulations such as those provided by the the Coupled Model Intercomparison Project phase 5 (CMIP5) have been extensively used to this aim. However, the spatial resolution of these simulations and the complex orography of the Canary Islands make it necessary the use of regional climate models operating at convection-permitting resolutions^26,27^ to accurately simulate the physical processes that govern climate in this territory.

The aim of this work is to project future changes in the climatic fire danger conditions and to analyze the weight of climate parameters in the expected changes. To this end, fire danger indices and regional climate projections are used. In particular FWI index and those related to it^16^ such as the Seasonal Severity Rating (SSR), the 90th percentile of FWI (FWI90), the percentage of days with an FWI greater than 30 (FOT30), and the extension in days of fire weather season (LOFS) have been used. The close relationship existing between the Canary Island pine and genuine Mediterranean pines^28^, the previous studies in the FWI applicability in Mediterranean area^29^, and the predominance of the subtropical climate in both regions, support the feasibility of using these indices in the Canary Islands. To estimate these indices under climate change conditions, the simulation results of three global climate models (GCMs) included in CMIP5, have been used as initial and boundary conditions for the Weather Research and Forecasting (WRF) model to simulate climate variables at two 30–year periods in the future: 2030–2060 y 2070–2100. These projections were carried out using two different greenhouse gas concentration pathways, the CMIP5 representative concentration pathway 4.5 and 8.5 (RCP4.5 and RCP8.5)^30,31^.

The present work is structured as follows: In the next section, the annual cycle of the selected danger indices are compared with the number of fires and the burnt area for the recent past period. Projected future changes across the archipelago are then analysed, and three specific sensitive points are studied, for which the weights of the different parameters are calculated. The “Methods” section presents the WRF model configuration, fire danger indices, statistical methods to assure the robustness of the projections, and the analysis of the relative influence of the different meteorological variables on FWI.

## Results and discussion

### Burnt area and FWI

**Figure 1:**
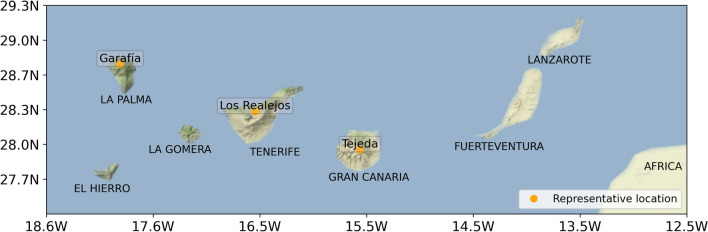
Location of Canary Islands and the three municipalities with the largest accumulated burnt areas during the study period. Map was created using the cartopy module version 0.18.0 (http://scitools.org.uk/cartopy/) for python 3.8.3 (http://www.python.org/). The terrain image was downloaded on demand from the Stamen tile server (http://stamen.com, under CC BY 3.0. Data by OpenStreetMap under ODbL).

The Spanish Area of Defense against Forest Fires Database contains a burnt area dataset by municipalities, which provides the date of fire detection and the extent of the burnt area from 1983 to 2009. In the analyzed period, 2150 wildfires were recorded in the Canary Islands, 93% of them did not exceed 10 ha. Fire incidence is limited to the five westernmost islands, since they are the most mountainous and more vegetated islands. Fuerteventura and Lanzarote, with very scarce vegetation, did not register fires. The three municipalities with the highest accumulated burnt area were selected to carry out more in depth studies. These are: Tejeda (Gran Canaria): 19,010 ha, Los Realejos (Tenerife): 17,409 ha, and Garafía (La Palma): 7845 ha; see locations in Fig. 1.

**Figure 2:**
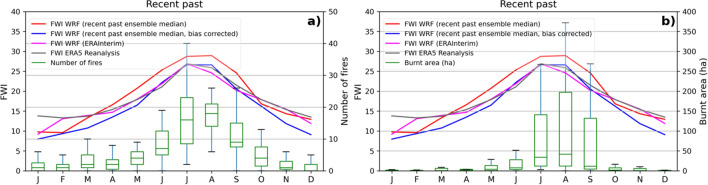
Monthly mean FWI of WRF median Ensemble in recent past period, 1980–2009 (red), bias corrected (blue), FWI computed from ERAInterim-WRF (magenta), and FWI ERA5 reanalysis (gray) (left scale). Annual cycle of the number of fires (**a**) and monthly burnt area (**b**) on the five western islands in recent past period (green box-plots, right scale). Boxes: monthly values of the lower quartile (Q1), median, and upper quartile (Q3). Whiskers extend from minimum to maximum monthly values.

To examine the relationship between FWI and wildfire episodes, these records were compared with the mean index obtained from simulations for the five western islands, in the recent past. The FWI values from the WRF simulations driven by each of the three GCM data (GFDL, IPSL, and MIROC) were averaged over the selected area and on a monthly basis, then the median of the three simulations was calculated (WRF ensemble). The annual cycle of the number of wildfires and monthly burnt area is shown in Fig. 2, together with monthly FWI. The vast majority of large fires have occurred during the summer months, since the burnt areas in July, August, and September account for 93% of the total burnt area, presenting a high inter-annual variability. As previously mentioned, in just one year, 2007, was burned 47% of the entire affected area during the 27 analyzed years. It is precisely in July and August that the monthly mean FWI is maximum. In addition, the annual cycle of the number of fires follows a similar behaviour to the mean FWI.

Furthermore, annual cycle of FWI computed from a WRF simulation (1995–2004) driven by Era-Interim data (ERAInterim-WRF)^26^, and FWI obtained from ERA5 reanalysis (1980–2009)^32^ are also presented to evaluate the model skills in reproducing FWI index for the recent past period when reanalysis or CMIP5 GCMs are used as boundary conditions. Despite the difference in the resolution of the two datasets obtained from reanalysis, approximately 5 km for ERAInterim-WRF and 25 km for ERA5, the annual cycle of the FWI averaged for the 5 western islands is remarkably similar. The FWI bias between WRF ensemble and ERA5 Reanalysis is generally less than ±4, which has been conveniently reduced with the bias correction applied, especially between June and October, corresponding to the fire season and the period preceding it. The results presented hereinafter have been previously bias adjusted with MBCn (Multivariate Bias Correction) methodology^33^. Therefore, in the recent past, the WRF ensemble is able to reproduce the seasonal variability. The main statistical results of the total spatial and temporal variability of the different simulations are summarised in Supplementary Fig. S1 online. The comparison with the fire regime leads to the conclusion that FWI projected by high-resolution models can be considered as a weather-driven fire danger predictor.

FWI90 index is also related to the number of fires (Supplementary Fig. S2 online). However, the monthly mean percentage of grid points with FWI > 30 (Supplementary Fig. S3 online) has a less smooth behaviour, with a more pronounced peak in the summer months, in accordance with the months in which the burnt area is larger. Threshold 30 is the most commonly used in the literature, although it may vary from one region to another. According to the data analyzed in the period 1995–2005 using ERAInterim-WRF, in the Canary Islands, more than 80% of the fires burning more than 30 hectares occur when the FWI in the affected area is higher than 30 (see Supplementary Fig. S4 online). For this reason, this threshold has been used in this study. Similarly, although only 30% of the fires occur when FWI > 60, they correspond to 70% of the total burnt area, therefore this threshold has been chosen to define the days of extreme fire risk.

### FWI projections

**Figure 3:**
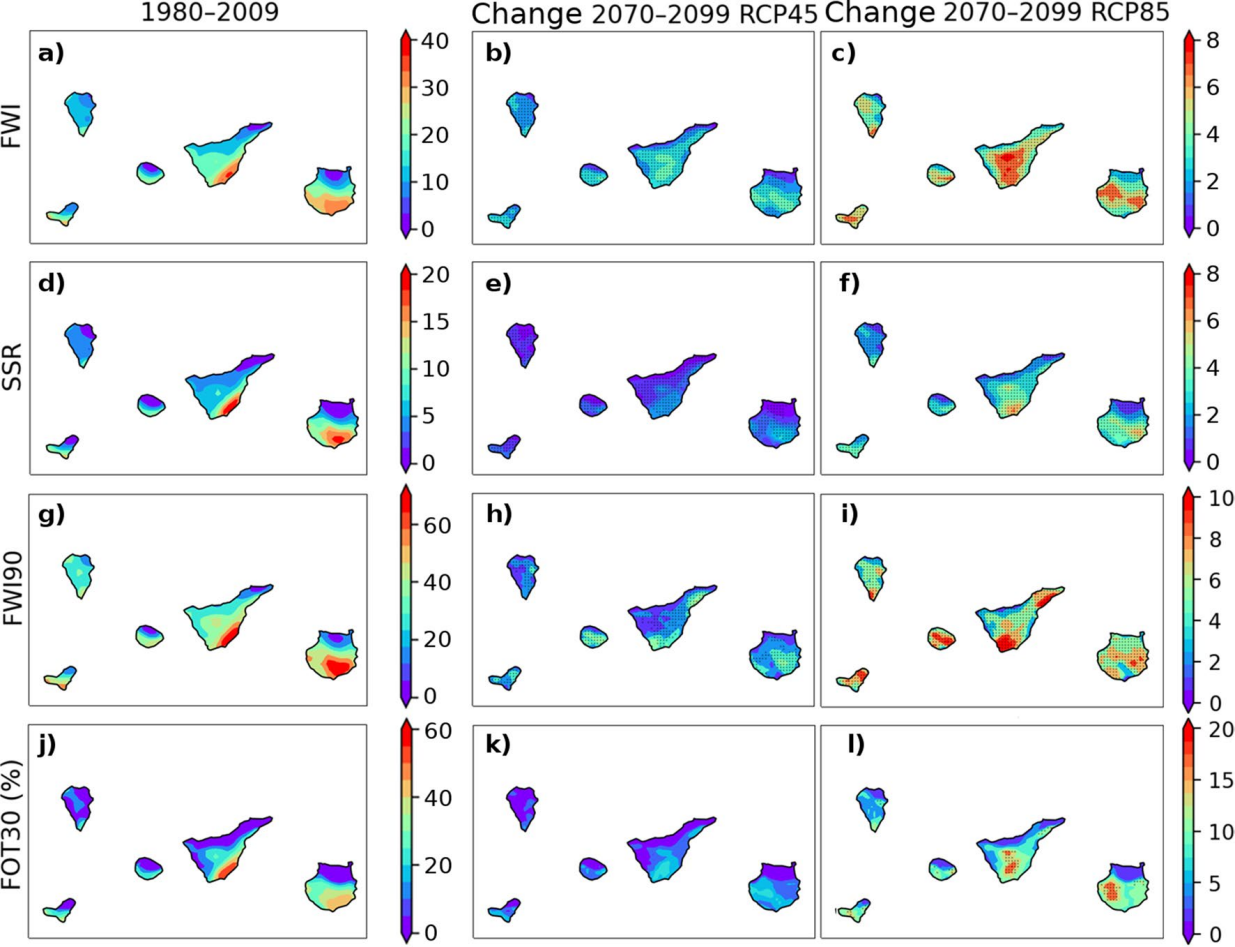
Evolution of the spatial distribution of the median values across three models of the annual means of FWI (**a**,**b**,**c**), SSR (Seasonal Severity Rating) (**d**,**e**,**f**), FWI90 (the 90th percentile of FWI) (**g**,**h**,**i**), and FOT30 (percentage of days with an FWI greater than 30) (**j**,**k**,**l**) indices, between recent past (1980–2009) (**a**,**d**,**g**,**j**) and future (2070–2099) under the two scenarios RCP4.5 (**b**,**e**,**h**,**k**) and RCP8.5 (**c**,**f**,**i**,**l**). Robust changes are marked with x.

All computed fire indices (FWI, SSR, FWI90 and FOT30) for the Canary Islands increase at the end of the century. As expected, the increase is much higher and statistically significant (see “Statistical methods” section) for the worst scenario, RCP8.5 (Fig. 3). Under both RCP4.5 and RCP8.5, at horizon 2070–2099, all expected changes are robust on most of the land. The percentage of land grid points with statistically significant changes is between 43 and 64%, depending on the index considered, for RCP4.5 projections. At the RCP8.5 scenario, the percentage varies between 79%, for LOFS, and 92%, for FWI. Conversely, changes in FOT30 are not statistically significant in the intermediate emissions scenario. The changes projected for all the indices in the mid-century period (2030–2059) have the same trend as those obtained for the end of the century, but they are not statistically significant in most of the territory, so they have been excluded from the final analysis.

In the recent past, there is a clear north-south pattern of FWI and its derived indices, across the islands. The archipelago is, during a considerable part of the year, under the influence of the trade winds, which blow from the northeast, bringing a flow of humid and cooler air. This fact, together with the relief and the semi-permanent subsidence inversion, is fundamental in the climatic differences between the south and the north of these islands. This dependence of FWI and the different meteorological variables on height and slope orientation for the island of Tenerife, which is the highest one, is summarized in Supplementary Fig. S5 online. In future projections, the difference between the drier and warmer south-facing leeward slopes and the north-facing slopes, which are more directly influenced by the humid trade winds, is preserved or even slightly increased. The projected change is more noticeable at higher altitudes due to the elevation-dependent temperature change and a remarkable decrease in precipitation. This behaviour of temperature and precipitation is consistent with previous studies in the same region^26^.

In a large part of the territory, as shown by the FWI90 values, 10% of the days the FWI index is above 20, and in a considerable percentage above 40. This also corresponds to the number of days with FWI>30, which is greater than 10% of the annual days in most of the surface of the islands.

Despite the small surface of the archipelago, the rugged terrain causes high spatial variability in the fire danger weather conditions projected in both climate change scenarios. Especially remarkable are the increases in altitude and under high-emission pathway; on average, FWI, SSR and FWI 90th percentile increase by 10%. The increase in FOT30 exceeds 10% in mountain areas, where the forests are currently located. In the most unfavorable scenario, not only is the average FWI higher, but also the variability is greater among the different locations (see Supplementary Fig. S6 online).

**Figure 4:**
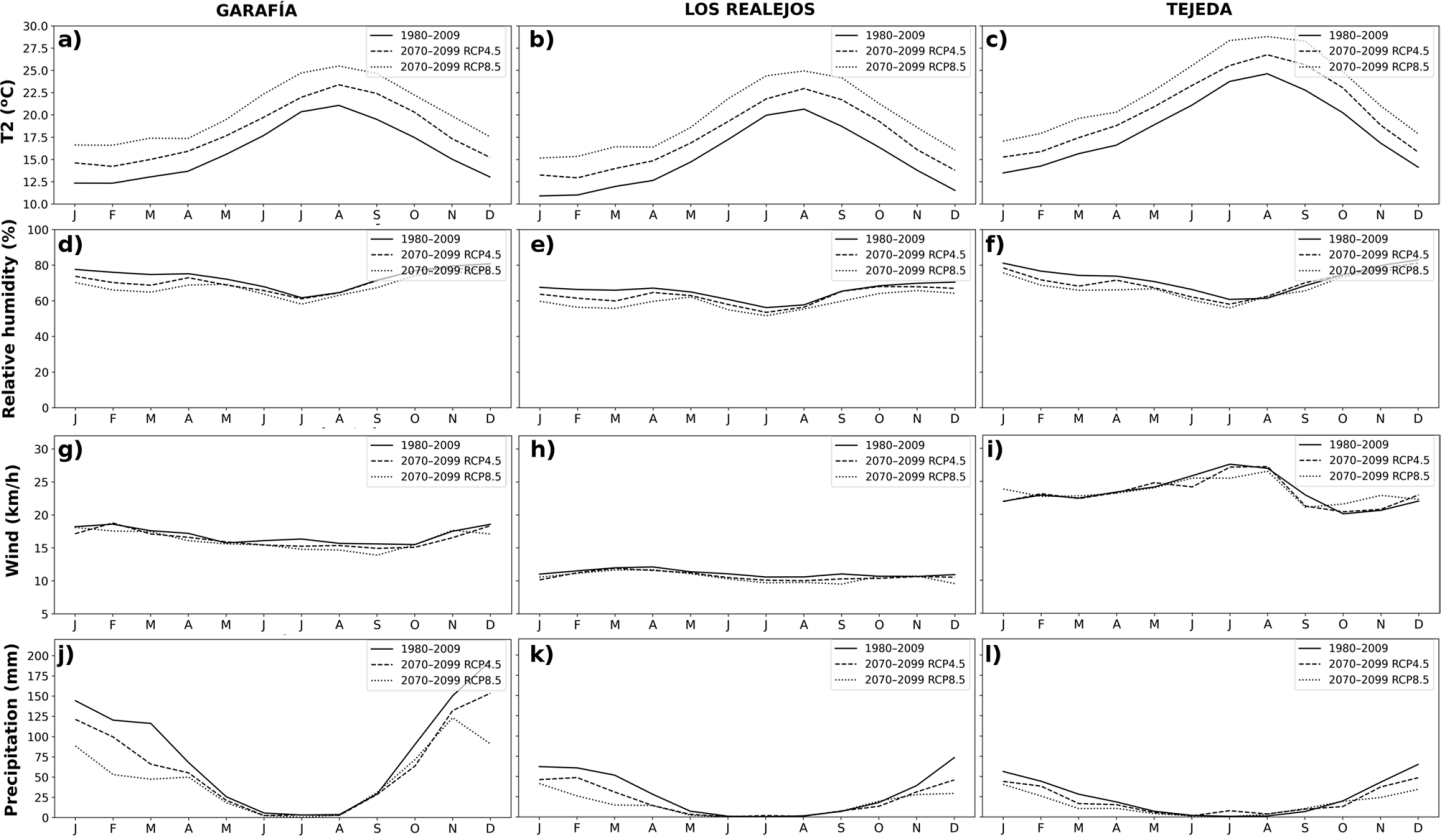
Monthly mean time series of 2 m temperature (**a**,**b**,**c**), 2 m relative humidity (**d**,**e**,**f**), 10 m wind speed (**g**,**h**,**i**) and accumulated precipitation (**j**,**k**,**l**), for the three selected locations and different 30 year periods simulations: recent past (1980–2009) and future (2070–2099), using scenarios RCP4.5 and 8.5.

To understand the changes projected in these indices, the variations in the different meteorological variables are analyzed in the selected locations. Monthly WRF ensemble values of these variables were computed on the three selected grid points, from west to east (Fig. 1): Garafía (1557 masl), Los Realejos (1804 masl), and Tejeda (1507 masl). Figure 4 presents simulated meteorological variables in the two periods of 30 years, recent past and future, in this last case for the two selected emissions scenarios. Annual cycles describe a strong increment in temperature, between 3.6 and 5.5 °C in the worst scenario, with little variations throughout the year, in agreement with the results obtained in previous studies^26,34^. March is the month with the greatest reduction in relative humidity, reaching 13% at RCP8.5. The wind does not show relevant changes in these locations, remaining these variations in the worst-case scenario within the range between − 2.1 and 2.3 km/h, consistent with previous studies^35,36^. Finally, the decrease in precipitation is remarkable during the winter months. Averaging the three locations analyzed, in the RCP4.5 scenario the reduction in the estimated accumulated precipitation is 23%, reaching 41% in the RCP8.5 scenario.

### Length of fire season and extreme values projection

**Figure 5:**
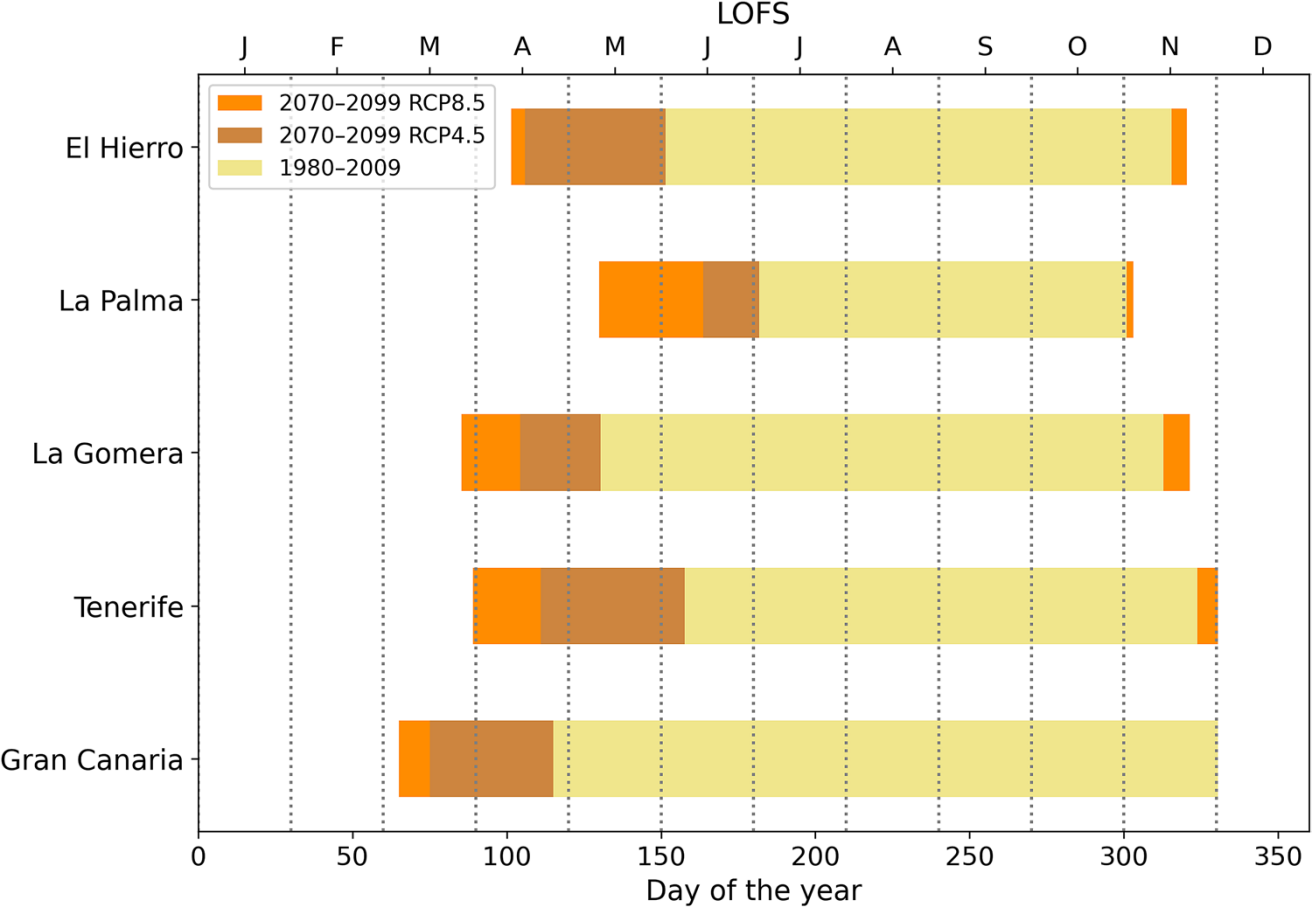
Length of the fire season (days) for different islands, indicated by the length of the horizontal bars. The vertical grid lines facilitate the identification of the initial and final day of the season in each period: recent past, 1980–2009 (beige), and future, 2070–2099, RCP4.5 (brown), 2070–2099, RCP8.5 (orange).

Estimates of fire season length for the recent past and the expected future changes are shown in Supplementary Fig. S7 online. It is currently very long in the southern areas of the islands, exceeding 300 days in the south of Tenerife and Gran Canaria. As these grid points correspond to the areas where the highest temperatures and the lowest humidity are recorded^37^, they are scarce in vegetation. As a consequence, the fire season predicted by the LOFS index can no longer increase in these zones. On the other hand, in the three westernmost islands and in the north of Tenerife and Gran Canaria, the currently length of the season is shorter, even below 150 days in many areas. Therefore, future changes are much more pronounced in this zone. The length of the fire season is summarized for each island in Fig. 5 (see numerical values in Supplementary Table S2 online). La Palma is the island with the shortest season, while Gran Canaria has the longest LOFS. The fire season coincides in all the islands between June and October, that corresponds to the months in which most of the fires occur (Fig. 2).

For both future scenarios, the fire weather season is predicted to be from 16 days longer in La Palma for the RCP4.5 scenario, up to 75 days in Tenerife under the RCP8.5. In this worst case scenario, the fire season on the island of Gran Canaria is expected to last 258 days, i.e., almost 9 months a year. Averaging the five western islands, climate models foresee an increment in the fire season at the end of the century of 14% and 33% in RCP4.5 and RCP8.5, respectively.

Tenerife is the most affected island by the worsening of this index. The explanation could be that it is the island with the highest elevation, reaching 3718 m on the Teide peak. These increases in LOFS are in line with other experiments^2,14,16^. Previous studies have also estimated a more pronounced future increase in temperature in the highest areas of the Canary Islands, as well as a reduction in precipitation and, therefore, in humidity^26^. The fire season is projected to spread at the beginning and end, although it is early in the spring when the largest increases are expected. More specifically, when averaged over the five islands, the beginning of the fire season is 35 days earlier in the RCP4.5 scenario and 53 days earlier in RCP8.5 at the end of the century, in comparison to the recent past.

To highlight the predicted changes in the upper tail of FWI, Table 1 shows, for each island, the annual mean of days with FWI > 60 and the ratio of grid points with FWI > 30. Both indices are projected to increase significantly across all islands and scenarios, even though the increment in the number of days with extreme fire weather danger conditions in the RCP4.5 scenario is statistically non-significant for three of the islands. In general, the average of the western islands shows an increase of 58% in extreme days, reaching 12 days a year in the worst scenario, and the area where the high values appear could increase by 44%, computed as the annual average, i.e., 22% of these islands area would be under high risk by the end of the century in the emissions scenario RCP8.5. Supplementary Table S3 online summarizes the same indices, in the recent past and projected into the future, averaged at different ranges of heights. Both, the number of days of extreme risk and the percentage of the land surface with FWI>30, substantially increase at the highest elevations, where the relative changes are almost twice those expected in the coastal areas.

**Table 1:** Projected averaged changes, for each island, in extreme fire risk days and percentage of surface with a high risk of fire.

Island	Time period	Extreme fire risk days	% surface at high risk
		(FWI > 60)	(FWI > 30)
El Hierro	1980–2009	6.7	14.6
	2070–2099 RCP4.5	7.7*	17.4
	2070–2099 RCP8.5	11.0	22.8
La Palma	1980–2009	1.7	7.9
	2070–2099 RCP4.5	2.1*	9.3
	2070–2099 RCP8.5	3.3	12.6
La Gomera	1980–2009	5.8	11.9
	2070–2099 RCP4.5	7.0*	14.2
	2070–2099 RCP8.5	9.7	18.0
Tenerife	1980–2009	5.9	13.5
	2070–2099 RCP4.5	7.4	15.8
	2070–2099 RCP8.5	10.1	19.9
Gran Canaria	1980–2009	13.2	21.7
	2070–2099 RCP4.5	15.4	24.7
	2070–2099 RCP8.5	19.3	29.5
Five Islands	1980–2009	7.4	15.0
	2070–2099 RCP4.5	8.9	17.3
	2070–2099 RCP8.5	11.7	21.6

*Statistically non-significant results, $P > 0.05$.

### Drivers of FWI in specific areas

**Figure 6:**
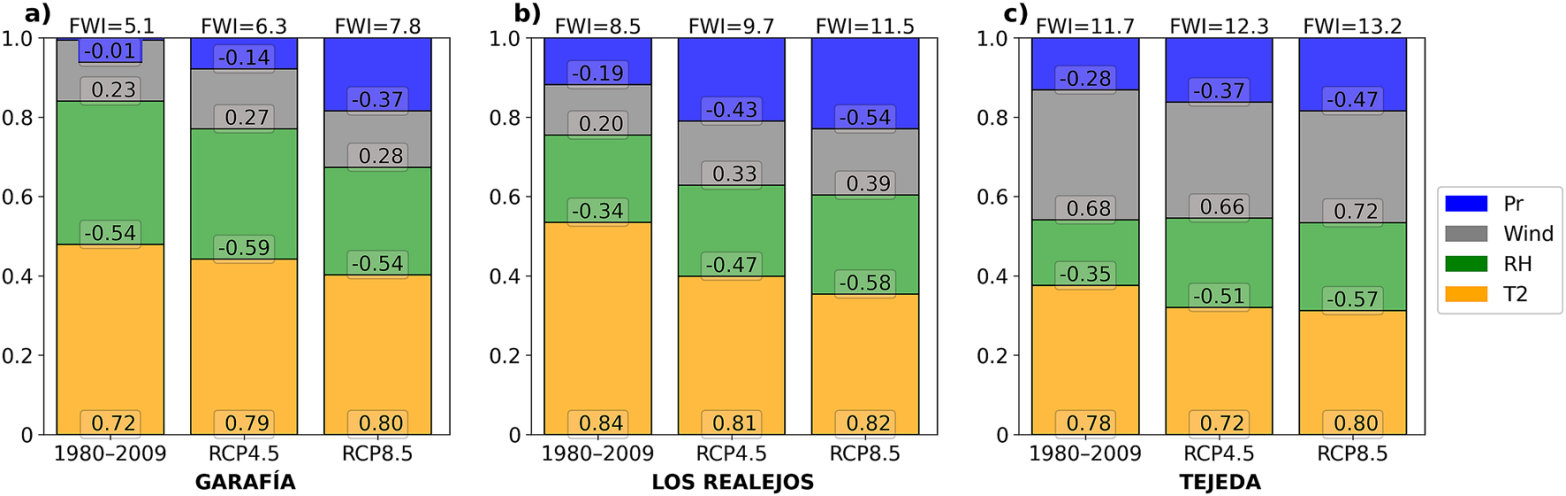
Partial correlation coefficients relative weights between FWI and parameters: 2 m temperature (T2), 2 m relative humidity (RH), 10 m wind speed (Wind), and precipitation (Pr), for 30 year projected simulations: recent past (1980–2009) and future (2070–2099), for scenarios RCP4.5 and 8.5. Partial correlations were calculated, for each selected location, from the time series of each of the models (GFDL-WRF, IPSL-WRF and MIROC-WRF) and the medians of these results are shown.

To evaluate the relative contribution of each parameter to the fire danger index, FWI, the partial correlation coefficients were computed for the three selected locations, and they are shown in Fig. 6, both for the recent past and the future periods. As expected, in all locations and scenarios, the partial correlations in temperature and wind are positive, and negative in relative humidity and precipitation. In the studied locations, temperature is the parameter with the greatest weight in the score of the fire index. However, its relative importance is expected to decrease in the future in the face of an increase in the weight of precipitation. Both relative humidity and wind would maintain their importance in the future. As an illustrative case, in Los Realejos the relative weight of temperature would reduce from 54% in the recent past to 35% at the end of the century in RCP8.5., while, on the contrary, precipitation would increase its weight within the FWI from the current 12% to 23%. Therefore, the reduction of precipitation will be increasingly important in the enhancement of the risk of fires.

## Conclusions

Wildland fire is a result of interactions between climate-weather, fuels and human factors. Despite important levels of vulnerability of small islands to climate change, future projection studies in these territories are still limited. This study aims to fill this gap in the Canary Islands, which are going to be particularly vulnerable to fires since previous studies foresee an increase in temperatures and drought. The results presented does not limit itself to the projection of the weather fire danger indices, but attempts to delve into the predominant factors that dominate the projected changes. Although temperature has a great weight in the risk of fires in the present and in the future, the projected decrease in rainfall will result in an increasing weight to this variable.

Weather variables projected with reliable regional climate models can explain the annual cycle of burnt areas in the recent past in the Canary Islands. Nonetheless, hindcast techniques may be implemented in the future to improve knowledge about other possible influences on wildfires, such as Saharan dust intrusions or different climate patterns^38,39^. The quantity of fuel is, on the other hand, critical to the intensity of the fires. It is not easy to predict how climate change will affect the amount of fuel. Decrease in future precipitation is directly related to the fire danger index increment in the future, but simultaneously should lead to a reduction in the bulk of fuel. Certainly, human populations have a high impact on the triggering of fires, through fire management, fire ignition with intentional or negligent behavior, and land-use^39,40^, that were not considered in this study.

Despite these limitations, we illustrate that at the end of the century it is projected a worsening in all fire danger indices studied, more pronounced, as expected, in the RCP8.5 scenario. The simulations for the islands indicate an increase in fire risk, length of the fire season, and the number of days with extreme risk. Although the general trend is towards a worsening of the indices, the expected future changes are highly variable from one island to another and even in different areas of the same island, which reinforces the need to carry out climate regionalizations with sufficient resolution in mountainous and/or fragmented territories.

An increase in the percentage of the territory of the islands with adverse fire weather indices is predicted by the end of the century. In addition to the direct costs of extinguishing fires or of the properties that may be affected, fires have acute effects on biodiversity and endemic species that are a visitor claim, in an economy highly dependent on the tourism sector^41^. Current fire management actions might not be sufficient to balance climate change consequences, and coupled atmosphere-wildfire would be useful to improve adaptation strategies.

To summarise, our results substantially align with previous studies in other regions that assessed the impact of climate change on fire risk increase, with further observing a reinforcement in height due to the great unevenness of most of the islands. Our findings support the statement of the Intergovernmental Panel on Climate Change that limiting the greenhouse gas concentration reduces the risk of wildfires.

## Methods

### Study region

The Canary Islands, located in the Macaronesia region of the North Atlantic Ocean next to the northwest coast of Africa, are a small territory, 7493 km$^{2}$, whose flora is rich in endemic species, being remains of the Tertiary Epoch of the Mediterranean Region and the drier regions of Africa, with a huge scientific value^42,43^. The five westernmost islands have an abrupt orography, exceeding 1400 m high. They contain a great part of the vegetation, mainly in their windward slopes. Between approximately 500 and 1200 masl, the broadleaf evergreen forest is located. Upslope, the pine forest can be found, reaching almost 2000 masl^44^. The endemic pine *Pinus canariensis* is the main forest species of the islands chain, occupying more than 60% of the total forest surface of the islands^45^, and one of the most resistant pines to mortality by fire, as a result of the islands volcanic origin^25^.

### Regional climate simulations

A dynamical downscaling technique has been applied, using the hydrostatic Weather and Research and Forecasting (WRF) model (WRF/ARW, v3.4.1)^46^. A one-way triple nesting setup was used, with spatial resolutions of 27, 9 and 3 km. The coarse outer domain, centered in the north-eastern Atlantic, covers a wide region to capture the main mesoscale processes that affect the Canary Islands, while the two innermost domains are centered in this archipelago (see Supplementary Fig. S8 online). The WRF version and the physical parameterizations used to represent the different sub-grid scale atmospheric processes were selected in previous studies in the same area^26,47^. Specifically, the selected physics schemes were the WRF double-moment 6-class (WDM6)^48^ microphysics scheme, the Yonsei University planetary boundary layer scheme^49^, the Noah land surface model^50^ and the Community Atmosphere Model version 3^51^ scheme, for both longwave and shortwave radiation. Regarding cumulus parameterization, the Kain-Fritsch scheme^52^ was used in the two outermost domains. The vertical resolution follows the same configuration as in^26,47^, with 32 vertical levels unevenly distributed, mainly concentrated in the lower part of the atmosphere.

The boundary conditions for WRF simulations were obtained from three of the models participating in CMIP5 (Table 2). From all the available realizations for each GCM, only the results corresponding to r1i1p1 were used. The simulations were performed for three different periods: recent past (1980–2009), mid (2030–2059) and end (2070–2099) of the 21st century. Actually, each period consists of 31 years, but the first year of each simulation was taken as the spin-up period and was excluded from the subsequent analyses, using only the last 30 years. For the future periods, two different greenhouse gas concentration pathways, the CMIP5 representative concentration pathway 4.5 and 8.5 (RCP4.5 and RCP8.5) scenarios were used, representing middle and high emission assumptions, that lead to stabilization of radiative forcing at 4.5 and 8.5 W m$^{-2}$ at the end of this century, respectively^53^. The reason for using only three models is the high computational cost of simulating, for each of them, 5 periods of 31 years, with a time step, for the innermost domain, of 12 seconds. The choice of the particular models, which is an important aspect in the design of any climate regionalization experiment, was based on previous studies for the African CORDEX domain^54^ and on the availability of the necessary data with adequate temporal resolution.

**Table 2:** CMIP5 GCMs used as initial and boundary conditions in the downscaling experiments.

GCM name	Institution	Resolution	Reference
GFDL-ESM2M	Geophysical Fluid Dynamics	2.5 × 2	^55^
	Laboratory, USA		
IPSL-CM5A-MR	Institut Pierre Simon	2.5 × 1.25	^56^
	Laplace, France		
MIROC-ESM	The University of Tokyo,	2.8 × 2.8	^57^
	National Institute for		
	Environmental Studies, and Japan		
	Agency for Marine-Earth		
	Science and Technology, Japan		

### Fire weather danger analysis

The FWI^13^ is based on the effects of weather parameters on forest floor fuel moisture conditions and generalized fire behavior in a standard jack pine stand. Nowadays, it is globally used as an indicator of fire risk. It requires the next variables as inputs: air temperature, relative humidity, and wind speed at 12:00 local standard time and 24 hours accumulated precipitation. The index is calculated from five components: three primary components representative of moisture content and two intermediate components representing the rate of spread and fuel consumption. The final FWI value, represents the intensity of the spreading fire as energy output rate per unit length of fire front, and it is widely used as a general index of fire danger^13–18^. Other more specific indices to calculate the impact of climate change on fires are inferred from the FWI:

- The Daily Severity Rating^13^ is calculated as an exponential function of FWI to better reflect the awaited efforts for fire suppression. It is generally averaged, seasonally, as Seasonal Severity Rating (SSR).
- FWI90 is the 90th percentile of FWI. High values of FWI90 indicate a 10% of days, during each year, with elevated fire risk. High values, that correspond to extreme events, can be considered over 45^14^. This index is useful to analyze the future evolution of fire risk in the days with the highest score values.
- FOT30 indicates the percentage of days with an FWI greater than 30. This value is usually considered as an indicator of severe fire danger situations^4,16,58,59^.
- LOFS index measures the extension in days of fire weather season. This starts/finishes each year when FWI exceeds/decreases the value 15 for more than two consecutive weeks^14,16^.
- Extreme fire risk days have been defined as those in which the FWI index exceeds the high rating of 60. Its future projection can be a strong indicator of the impact of climate change on the number of days with weather conditions which make the territory extremely vulnerable to fires.
- Percentage of surface at high risk, defined as the area with FWI greater than 30. A future increase would indicate the impact of climate change on the percentage of the territory exposed to wildfires.

### Bias adjustment

Despite the continuous increase in spatial resolution and improved parameterization of physical phenomena in regional climate models, the results of simulations often remain biased compared to observations^60^. These errors are partly inherited from the driving global climate models. Therefore, especially in results relevant to impact studies, some method of bias adjustment should be applied. In the case of climate indices, such as the FWI, which are calculated from some essential variables provided by the models, there is a debate on the convenience of applying a bias correction method to each of these variables or to the resulting index directly^61^. In this study, a component-wise adjustment has been used, since an analysis of the present and future influence of each of these components on the overall index is carried out. Specifically, the multivariate bias correction (MBCn) algorithm^33^ was applied, using the MSBK (Statistical Bias Correction Kit) module (https://github.com/yrobink/SBCK) for Python. It is a multivariate generalization of quantile mapping that transfers the statistical properties of an observed continuous multivariate distribution to the corresponding distribution of simulated variables. Thus, the method is applied to all four variables (temperature, precipitation, humidity, and wind speed) at the same time, not to each of them separately, taking into account the dependence between them. The method, which uses a trend preserving algorithm, demonstrated its ability to adjust the bias in the FWI calculation over a North American domain^33^.

The ERAInterim-WRF simulation was taken as the reference data to adjust the bias of the simulations driven by GCMs in both the recent past and future periods. To overcome the mixed discrete-continuous nature of daily precipitation data, dry days were treated as censored values below a trace amount of 0.05 mm^62^. Zeros in the reference and biased data were replaced with nonzero uniform random values below 0.05. After bias correction, those values were set again to zero. To avoid discontinuities at the transition between months, bias adjustment was applied to the central calendar month of a sliding three month window for each land grid point.

### Statistical methods

In order to verify that the changes in the mean future values are statistically significant, the monthly time series of the analyzed indices are calculated for each of the grid points. For those indices calculated for an island or an entire region, the time series correspond to the monthly means of the spatial averages. The one-tailed null hypothesis H0 of no difference or negative change in each of those points is tested. Since we cannot guarantee that each of the samples to be compared belong to a normal distribution, or other parametric distribution, or that they have a similar spread, a bootstrap procedure is applied^63^ to the corresponding recent past and future series, generating 1000 bootstrap samples using random re-sampling with replacement. In the specific case of the FOT30 (FWI > 30) index, which measures the proportion of days that exceed this score value, it has been considered more appropriate to apply a test of proportions (Chi-square) to analyze the statistical significance^64^.

At each grid point the future changes are considered to be robust if they are statistically significant (p-value < 0.05) for the three driving GCM data (GFDL IPSL MIROC), using the corresponding bootstrapping procedure or test of proportions, and if these three projected changes have the same sign (all positive or all negative)^65^.

### Influence of the different meteorological variables

The purpose of this analysis is to determine which input parameters exert most influence on the FWI: temperature, relative humidity, wind, and/or precipitation. The calculation of FWI is not carried out by means of an expression in analytical form, but depends on several sub-indices in which conditional expressions are used depending on the values of the meteorological variables. In addition, the sub-indices for a given day depend not only on the value of the inputs for that day, but also on their own values on the previous day, creating a memory effect. All of this makes the influence of meteorological variables on FWI strongly monotonic yet highly nonlinear^66^. Therefore, the analysis cannot be performed by direct methods, such as those based on differential analysis. Different statistical techniques have been used in previous studies, such as partial derivatives with all input parameters initially set equal to their 95th percentiles^15^, one-at-a-time sensitivity measures^19^, where repeatedly vary one parameter at a time while holding the others fixed, or partial correlation coefficients. The partial rank correlation coefficient is widely utilized for sensitivity studies^67–69^, and has been selected for this study. Before the partial correlation analysis was conducted, the data have been detrended to avoid spurious effects^67^. The correlation coefficients have been measured in the recent past and the future. This allow us to anticipate the expected future variation of the parameter weights.

## Supplementary Information

Supplementary Information.
