# Supplementary material for: Projections of wildfire weather danger in the Canary Islands
Source: Sci Rep. 2022 May 16;12:8093. doi: 10.1038/s41598-022-12132-5 (PMC9110393; doi:10.1038/s41598-022-12132-5)
Supplement: Supplementary file 1 — Supplementary Information. [file 41598_2022_12132_MOESM1_ESM.pdf]

Supplementary material

Projections of wildfire weather danger in the Canary Islands

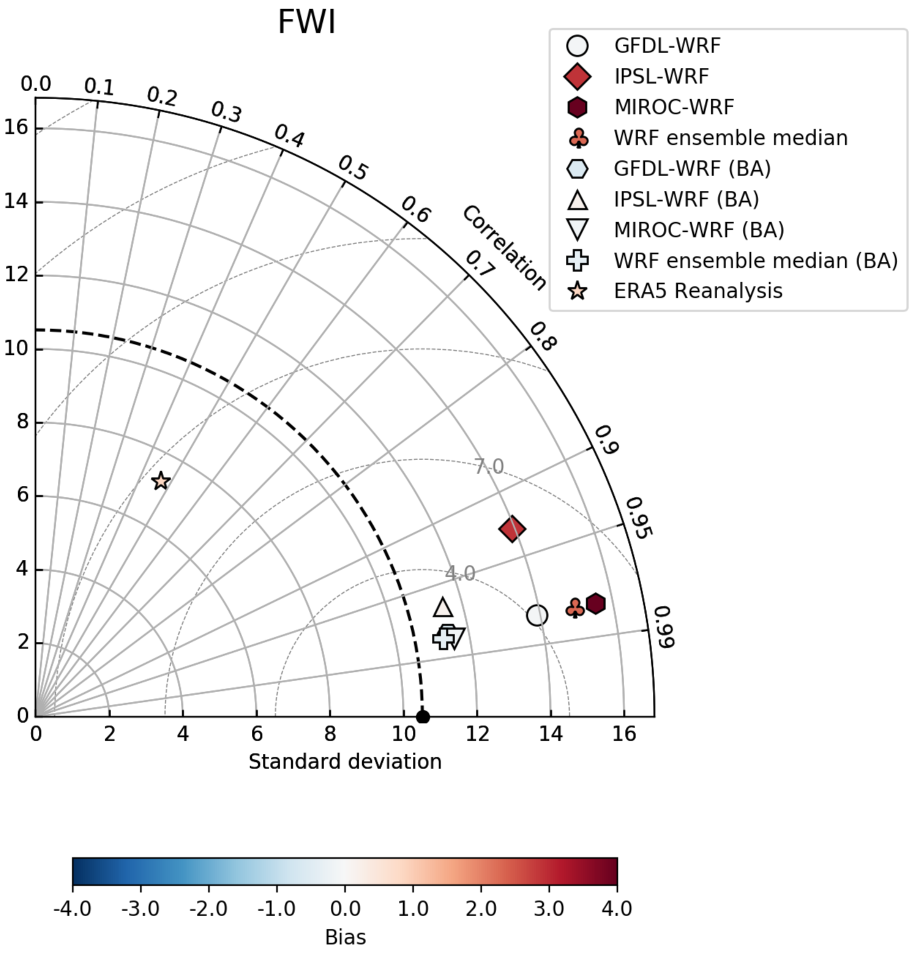

Supplementary Figure S1. Taylor diagram[1] illustrating the comparison between the different simulated data. The standard deviation of the monthly mean FWI computed from WRF simulation driven by ERAInterim data (ERAInterim-WRF) is represented by a solid circle on the abscissa. The other symbols, which represent the FWI data from ERA5 and the three climate WRF simulations for the recent past period, are positioned such that their standard deviation is the radial distance from the origin, their correlation coefficient with respect to the ERAInterim-WRF FWI is the cosine of the azimuthal angle, and their centred root-mean-square (CRMS) difference is the distance to the point on the abscissa. The corresponding biases are indicated in the legend. The spatial resolution of simulations driven by the global models (GFDL, IPSL and MIROC) is 3 km, that of ERAInterim-WRF is 5 km and that of ERA5 is about 25 km. Therefore all data were interpolated, using the nearest neighbor, to the ERAInterim-WRF grid, used as reference. All statistics are calculated for monthly averages of all land grid points. The correlations between the three highest resolution simulations and ERAInterim-WRF are above 0.9. The correlation of ERA5 is lower, due to its lower spatial resolution, although its bias is very small. The standard deviations are also higher in those simulations with higher spatial resolution. GFDL-WRF is the simulation with the lowest bias, with MIROC-WRF having the largest difference with respect to ERAInterim-WRF. The median of the ensemble of the three simulations has a bias of 2.2. The results of the application of the MBCn[2] bias adjustment (BA) method to the three climatic regionalizations are also shown in the diagram. The bias adjustment provides noticeable improvements for the three simulation results and, therefore, for the ensemble median.

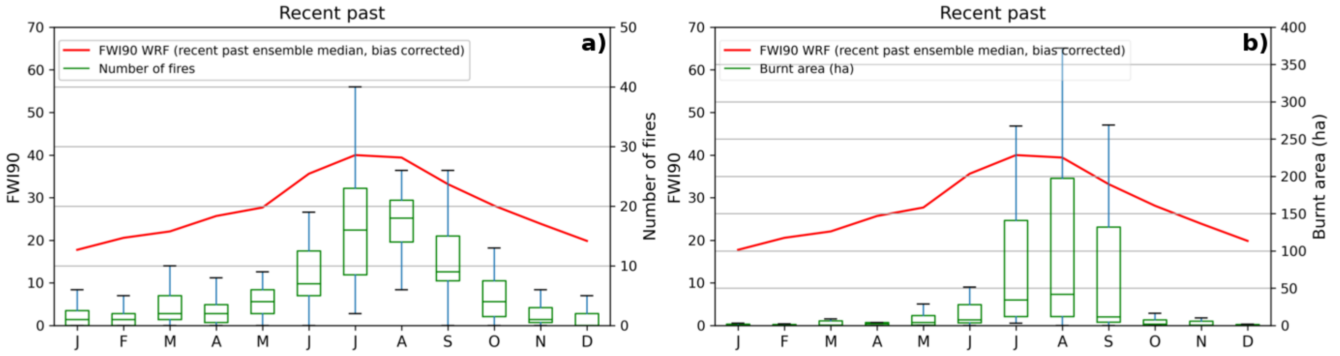

Supplementary Figure S2. Monthly mean FWI 90th percentile (FWI90) of WRF ensemble in recent past period (red, left scale). Annual cycle of the number of fires (a) and monthly burnt area (b) on the five western islands in recent past period (blue-green box-plots, right scale). Boxes: monthly values of the lower quartile (Q1), median, and upper quartile (Q3). Whiskers extend from minimum to maximum monthly values.

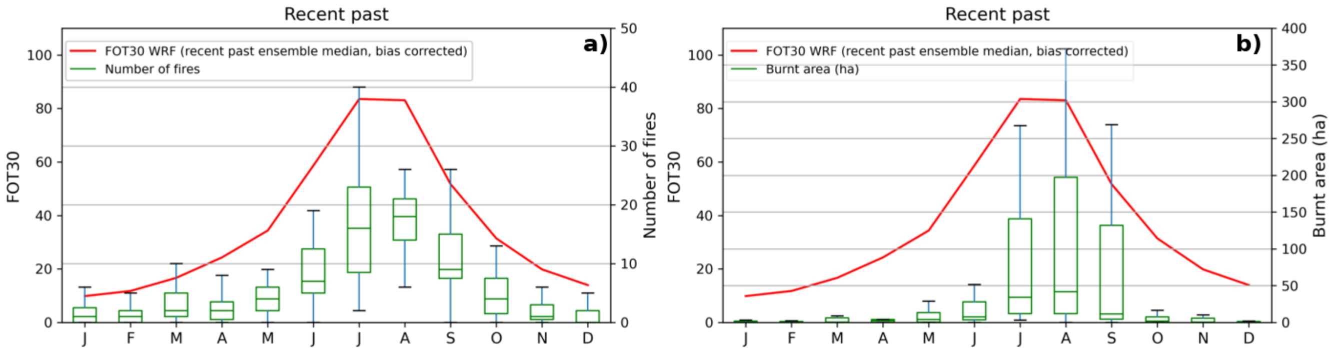

Supplementary Figure S3. Monthly mean percentage of gridpoints with FWI > 30 of WRF ensemble in recent past period (red, left scale). Annual cycle of the number of fires (a) and monthly burnt area (b) on the five western islands in recent past period (blue-green box-plots, right scale). Boxes: monthly values of the lower quartile (Q1), median, and upper quartile (Q3). Whiskers extend from minimum to maximum monthly values.

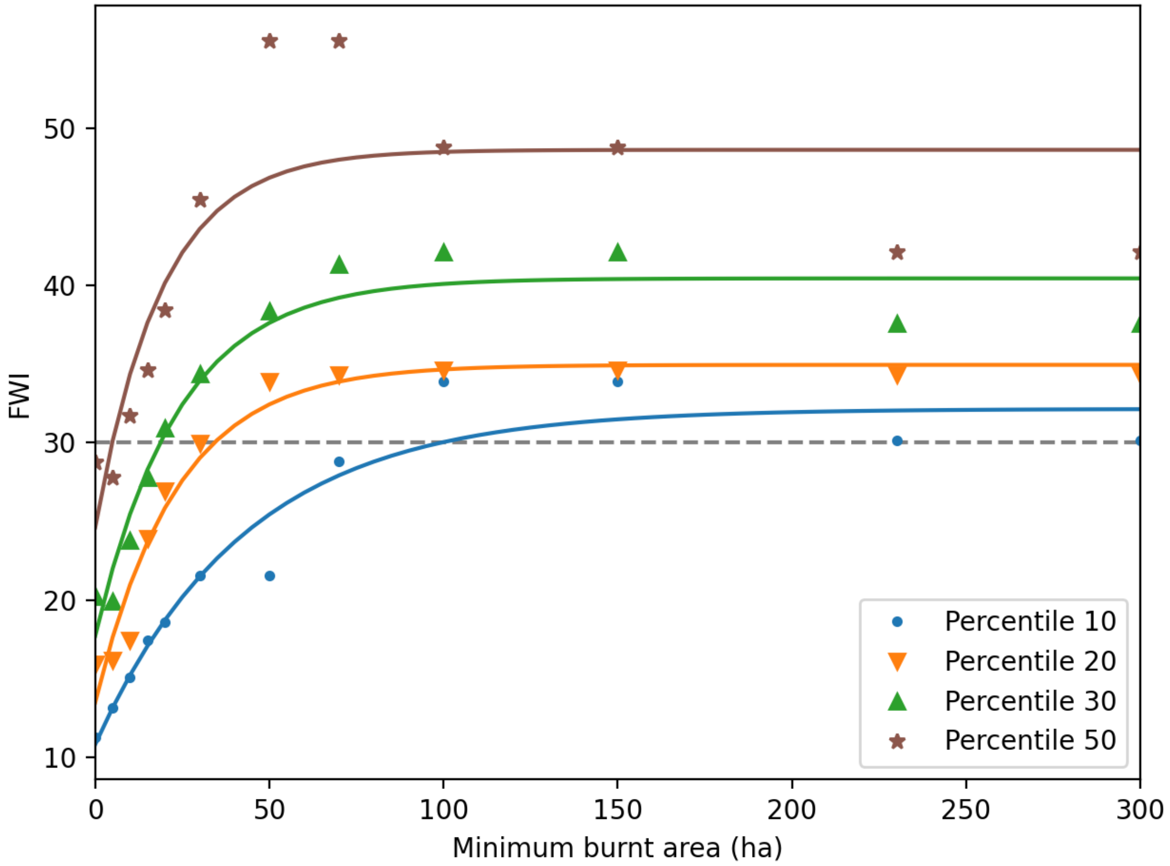

Supplementary Figure S4. FWI percentile curves for all fires that occurred in the Canary Islands between 1995 and 2004, 684 in total, with areas greater than those indicated on the abscissa axis. The FWI estimates correspond to the ERAInterim-WRF simulation and are calculated as the mean of the values at the grid points corresponding to the affected area in each wildfire. Thus, for example, observing the 20th percentile curve, it can be seen that 80% of wildfires with burnt area larger than 30ha, occur when FWI>30. Likewise, 90% of the fires with a burned area of more than 100 hectares correspond to FWI values above 30 (10th percentile curve).

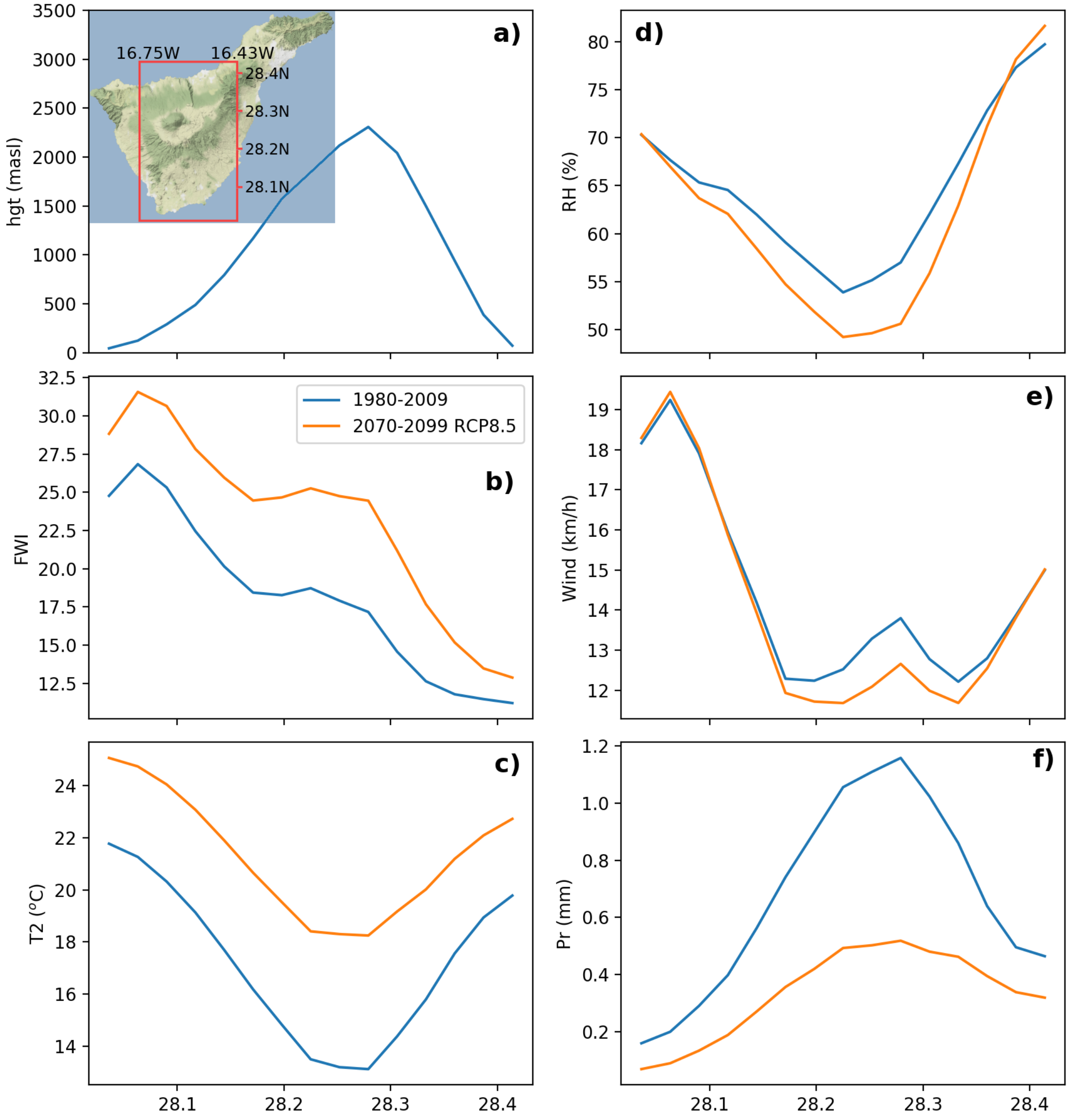

Supplementary Figure S5. Zonal average, corresponding to the transect indicated in the map, of annual means of FWI and the four related meteorological variables for two periods: recent past (1980–2009) and end of the century (2070-2099) RCP8.5. The shown values correspond to the mean of the three simulations, driven by the three GCMs. (a) hgt indicates terrain height (masl), (b) FWI is the fire weather index, (c) T2 is 2m temperature (^{o}C), (d) RH is relative humidity (%), (e) Wind speed is in units of km/h and (f) Pr is 24-hour accumulated precipitation (mm). The north-facing slopes are cooler and more humid, influenced by the semi-permanent trade winds. Precipitation is also higher on these slopes, causing the FWI to exhibit lower values. Projected future changes are greater in the southern zone, in the lee of the trade winds and, especially, in the upper areas, mainly due to a larger increase in temperature and a stronger decrease in precipitation.

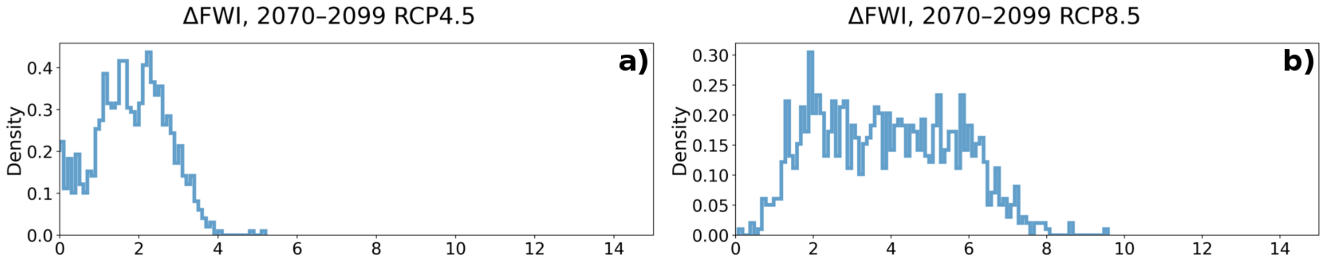

Supplementary Figure S6. Histograms of the annual mean FWI change calculated as difference between future (2070–2099) scenarios RCP4.5 (a) and 8.5 (b) and the recent past period (1980–2009), using WRF-Ensemble projections. The histograms are calculated from the values of each of the land grid points.

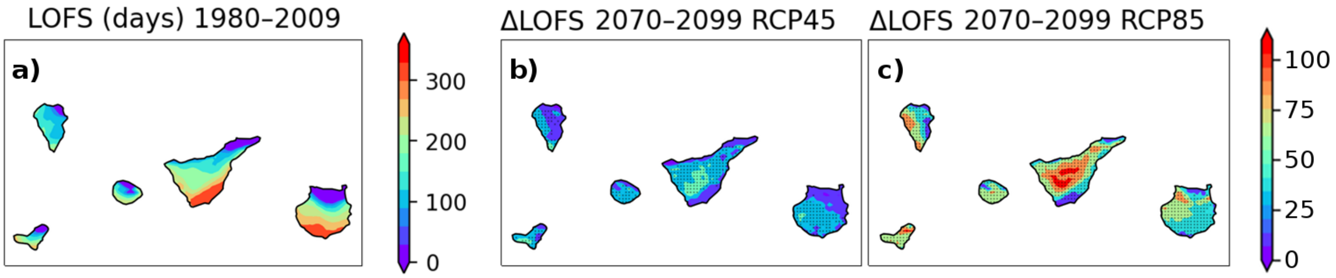

Supplementary Figure S7. Evolution of the spatial distribution of the median values across three models of the annual means of LOFS (extension in days of fire weather season) index, between recent past (1980–2009) (a) and future (2070–2099) under the two scenarios RCP4.5 (b) and RCP8.5 (c). Robust changes are marked with x.

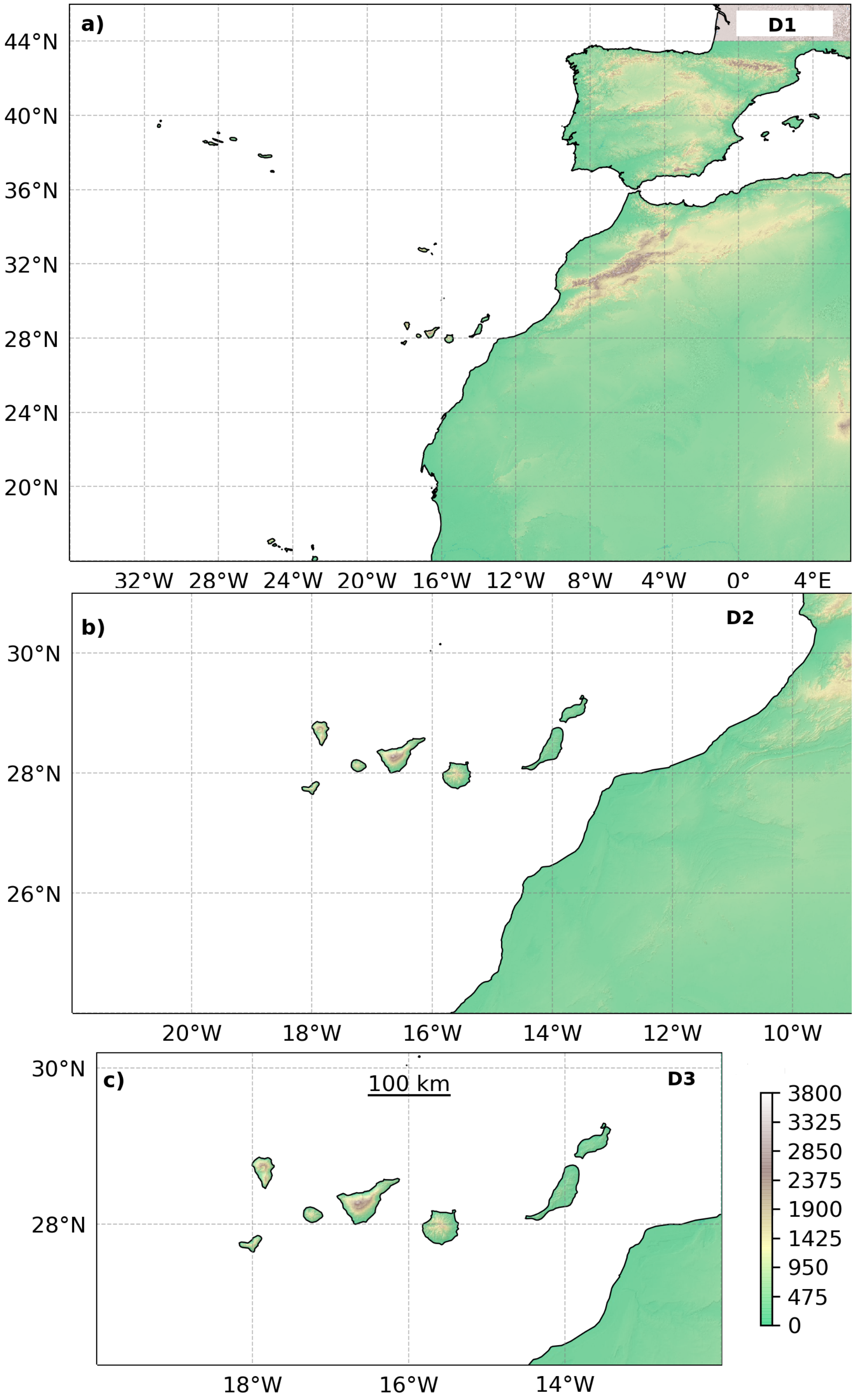

Supplementary Figure S8. Domains used in the WRF simulations. The coarse domain D1 (a) has an horizontal resolution of 27 km, the intermediate domain D2 of 9 km (b), and the innermost domain D3 (c) a resolution of 3 km. Land surface height (m asl) is indicated in the color palette to highlight the complex orography of the studied region. Maps were created using the cartopy module version 0.18.0 (`http://scitools.org.uk/cartopy/`) for python 3.8.3 (`http://www.python.org/`). Land topography was obtained from ETOPO1 1 arc-minute global relief model (`http://www.ngdc.noaa.gov/mgg/global/`).

Supplementary Table S1. Mean annual burnt area (1983–2009) of five EU-Med countries (France, Greece, Italy, Portugal, Spain), which account for around 80% of the burnt area of the 17 European countries analyzed in a previous study[3], and Canary Islands. The corresponding percentages have been calculated with respect to the total area of the countries or the archipelago. Burnt area data for Canary Islands have been provided by Spanish Government (Ministerio para la Transición Ecológica y el Reto Demográfico). The data for the European countries were obtained from the European Forest Fire Information System (EFFIS) database[4]. The total areas of the countries and the archipelago were obtained from Eurostat (https://ec.europa.eu/eurostat).

Country or archipelago	Mean annual burnt area (ha)	% area of the country/archipelago burned each year
France	22,900	0.04
Greece	48,250	0.37
Italy	93,068	0.31
Portugal	99,972	1.08
Spain	151,499	0.30
Canary Islands	2,826	0.38

Supplementary Table S2. Estimated day of the year of the beginning of the fire weather season and its extension in days (LOFS) in recent past: 1980–2009, and future: 2070–2099, RCP4.5, 2070–2099, RCP8.5 for the different islands.

Island	Time period	Day of year of the beginning	LOFS (days)
El Hierro	1980–2009	151	164
	2070–2099 RCP4.5	106	188
	2070–2099 RCP8.5	101	219
La Palma	1980–2009	182	119
	2070–2099 RCP4.5	164	135
	2070–2099 RCP8.5	130	173
La Gomera	1980–2009	130	182
	2070–2099 RCP4.5	104	205
	2070–2099 RCP8.5	85	236
Tenerife	1980–2009	158	166
	2070–2099 RCP4.5	111	200
	2070–2099 RCP8.5	89	241
Gran Canaria	1980–2009	115	215
	2070–2099 RCP4.5	75	236
	2070–2099 RCP8.5	65	258

Supplementary Table S3. Projected averaged changes by altitude intervals in extreme fire risk and fire weather affected area. All results are significant, $P < 0.05$. The increase in the number of days with extreme fire risk conditions due to weather influences, and the percentage of area with FWI>30 substantially increases at high altitudes.

Altitude	Time period	Extreme fire risk days (FWI > 60)	High risk % affected area (FWI > 30)
0–400m	1980–2009	8.7	16.1
	2070–2099 RCP4.5	10.4 (+20%)	18.1 (+12%)
	2070–2099 RCP8.5	13.4 (+54%)	21.5 (+33%)
400–1100m	1980–2009	7.8	14.8
	2070–2099 RCP4.5	9.2 (+18%)	17.4 (+18%)
	2070–2099 RCP8.5	12.0 (+54%)	22.1 (+49%)
> 1100m	1980–2009	3.4	12.3
	2070–2099 RCP4.5	4.5 (+32%)	15.0 (+22%)
	2070–2099 RCP8.5	6.7 (+97%)	20.8 (+69%)

References

1. Taylor, K. E. Summarizing multiple aspects of model performance in a single diagram. J. Geophys. Res. Atmospheres **106**, 7183–7192, DOI: 10.1029/2000JD900719 (2001).

2. Cannon, A. J. Multivariate quantile mapping bias correction: an n-dimensional probability density function transform for climate model simulations of multiple variables. Clim. Dyn. **50**, 31–49, DOI: 10.1007/s00382-017-3580-6 (2018).

3. De Rigo, D., Liberta, G., Durrant, T., Artes, V. T. & San-Miguel-Ayanz, J. Forest fire danger extremes in Europe under climate change: variability and uncertainty. Tech. Rep. KJ-NA-28926-EN-N, European Union, Luxembourg (Luxembourg) (2017). DOI: 10.2760/13180.

4. Camia, A., Durrant, T. & San-Miguel-Ayanz, J. The European Fire Database: technical specifications and data submission. Technical guidance LB-NA-26546-EN-N, Luxembourg (Luxembourg) (2014). DOI: 10.2788/2175.
